# The Observation Report of Red Blood Cell Morphology in Thailand Teenager by Using Data Mining Technique

**DOI:** 10.1155/2014/493706

**Published:** 2014-02-13

**Authors:** Sarawut Saichanma, Sucha Chulsomlee, Nonthaya Thangrua, Pornsuri Pongsuchart, Duangmanee Sanmun

**Affiliations:** Division of Clinical Microscopy, Faculty of Medical Technology, Huachiew Chalermprakiet University, Samut Prakan 10540, Thailand

## Abstract

It is undeniable that laboratory information is important in healthcare in many ways such as management, planning, and quality improvement. Laboratory diagnosis and laboratory results from each patient are organized from every treatment. These data are useful for retrospective study exploring a relationship between laboratory results and diseases. By doing so, it increases efficiency in diagnosis and quality in laboratory report. Our study will utilize J48 algorithm, a data mining technique to predict abnormality in peripheral blood smear from 1,362 students by using 13 data set of hematological parameters gathered from automated blood cell counter. We found that the decision tree which is created from the algorithm can be used as a practical guideline for RBC morphology prediction by using 4 hematological parameters (MCV, MCH, Hct, and RBC). The average prediction of RBC morphology has true positive, false positive, precision, recall, and accuracy of 0.940, 0.050, 0.945, 0.940, and 0.943, respectively. A newly found paradigm in managing medical laboratory information will be helpful in organizing, researching, and assisting correlation in multiple disciplinary other than medical science which will eventually lead to an improvement in quality of test results and more accurate diagnosis.

## 1. Introduction

Data mining technique is a process of discovering pattern of data. The patterns discovered must be meaningful in that they lead to some advantage. The overall goal of the data mining process is to extract information from a data set and transform it into an understandable data in order to aid user decision making. It utilizes methods such as statistics and mathematics to explore a relationship of data set or suitable conditions of those data, which leads to the extract of needed information or knowledge of relations. The decision tree is a supported modeling that represents the classification process of input data as a tree-like graph. It is based on Divide and Conquer concept, which is formed by many rules that branched out from the tree until the decision is made. There are many methods of decision tree algorithm such as AD-Tree, C4.5 decision (J48), or Random-Tree. The J48 algorithm is an open source JAVA implementing C4.5 in WEKA (Waikato Environment for Knowledge Analysis) software. The tree is constructed from gain ratio, the element with a highest gain ratio assigned as the root and uses gain ratio as the splitting branch of the tree [1].

The data mining has been used widely in many fields such as marketing, public relations, prediction of economy, and weather forecast. In hematology laboratory, it has become a powerful tool in managing uncountable laboratory information in order to seek knowledge that is underlying or within any given information. Many applications of data mining in hematology were proposed such as evaluated risk factors and relationship with life-threatening infection in children with febrile neutrophilia [2], created diagnosis approached to polycythemia vera [3], and proposed an original method to identify the immunophenotypic signature of chronic lymphocytic leukemia [4].

The authors applied data mining technique to dismiss bias from individual skill which makes the report very subjective. The relationships between red blood cell morphology reporting and hematological parameters (WBC, RBC, Hb, Hct, MCV, MCH, MCHC, PLT, NEU, LYMP, MONO, EO, and BASO) from blood cell analyzer were investigated. This study shows that by applying data mining, using hematological parameters from automated blood cell analyzer can help predicting the abnormality of RBC morphology as good as the RBC morphology which reported by individual skill. In the future, this guideline can be used as tools for laboratory improvement.

## 2. Material and Method

### 2.1. Sample and Data Set

The retrospective study used 1362 results from teenagers (17–19 years old) first-year undergraduate student checkup at Huachiew Chalermprakiet University in 2011. The data set included sex, hematological parameters, and RBC morphology. The hematological parameters from automated blood cell analyzer are composed of WBC, RBC, Hb, Hct, MCV, MCH, MCHC, PLT, NEU, LYMP, MONO, EO, and BASO (SysMex XT1800i, Sysmex corporation, Kobe, Japan). The peripheral blood smear was prepared and stained by ICSH standard protocol [5]; RBC morphology was manually evaluated by medical technologist who has a license certification from the medical technology council of Thailand. Collected data are assigned to two groups: normal RBC morphology and abnormal RBC morphology. The peripheral blood smears that are reported as normochromic and normocytic are categorized as normal RBC morphology while others fall into abnormal RBC morphology category (more details about data set are shown in Table 1).

### 2.2. Data Analysis by Data Mining Technique

The data mining analysis was analyzed by using WEKA version 3.6.9 which the collection of machine learning algorithms for data mining tasks [1]. The J48 which ones of decision tree of data mining technique was approached to this study. The evaluation of all the classifiers accuracy used a ten-fold cross-validation. The performance evaluation was averaged from all of ten separated evaluations. True positive (TP) was the number of abnormal RBC morphology predicted to be abnormal RBC morphology. False negative (FN) was the number of abnormal RBC morphologies predicted to be normal RBC morphology. True negative (TN) was the number of normal RBC morphologies predicted to be normal RBC morphology. False positive (FP) was the number of normal RBC morphologies predicted to be abnormal morphology. The validation measurements were investigated by accuracy, sensitivity, and specificity of result when compared with RBC morphology report. We focus on the following validation measures:

 Precision = TP/(TP + FN),
 Specificity = TN/(TN + FP),
 Recall = TP / (TP + TN),
 Accuracy = (TP + TN)/(TP + TN + FN +FP),
 
*F*-measure = 2 ∗ Precision ∗ Specificity/(Precision + Specificity).

### 2.3. Statistics

All data were presented as the mean ± standard deviation (SD). The significant evaluation between different categories was performed with the independent *t*-test. The *P* value less than 0.05 was considered as statistically significant.

## 3. Results

The hematological parameters from blood cell analyzer and RBC morphology report of 1362 cases were evaluated. There are 260 male cases (19.1%) and 1102 female cases (80.9%). The 1362 cases of RBC morphology were evaluated. Abnormal RBC morphology was found in 354 cases (25.99%) which classified as male 36 cases (2.64%) and female 318 cases (23.35%). The WBC, RBC, Hb, Hct, MCV, MCH, MCHC, and PLT of abnormal RBC morphology cases were significant from the normal RBC morphology cases in both male and female (*P* < 0.05). In the contrast to NEU, LYMP, MONO, EOS, and BASO were not significant RBC morphology (more details are shown in Table 2).

**Figure 1:**
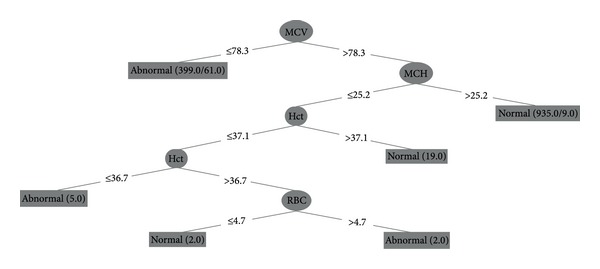
The decision tree from J48 algorithm in predicting RBC morphology. If MCV is less than or equal to 78.3 fL, RBC is labeled as abnormal RBC morphology but if MCV is more than 78.3 fL and MCH is more than 25.2 pg, RBC is still labeled as normal. And if MCH is less than or equal to 25.2 pg but Hct is greater than 37.1%, RBC is normal. On the other hand, if Hct is less than or equal to 36.7%, RBC is abnormal. But if Hct is between 36.7 and 37.1% and RBC is less than or equal to 4.7 × 10^12^ cell/L, it will most likely be normal RBC morphology. However, if RBC is more than 4.7 × 10^12^ cell/L, it is abnormal RBC morphology.

The data were analyzed by using J48 algorithm. The performance of J48 algorithm was evaluated by TP, FP, precision, recall, *F*-measure, and accuracy. The average RBC morphology prediction has TP, FP, precision, recall *F*-measure, and accuracy of 0.940, 0.050, 0.945, 0.940, 0.941, and 0.943, respectively. Interestingly, when all 13 data sets were analyzed by the algorithm, the program dismissed all but 4 data sets (MCV, MCH, Hct, and RBC) which were useful in predicting RBC morphology. According to the decision tree, if the MCV is less than or equal to 78.3 fL, it was labeled as abnormal RBC morphology. On the other hand, the MCV of greater than 78.3 fL, the interrelationship of MCH, Hct, and RBC are considered. In addition, this decision trees of both male and female show similarity which means that the decision tree was sex independent (more details were shown in Tables 3 and 4 and in Figure 1).

## 4. Discussion

The RBC morphology report is common, basic, and fundamental in hematology testing. Thus, it is needed in screening red blood cell abnormality before investigation into more specific diseases. RBC morphology reports in hematology laboratory are done manually. Undoubtedly, this report takes time, and at the end, it is very subjective and varies from one technologist to another due to individual laboratory skill and decision making skill.

The authors studied the mean and SD of parameters and found that no parameter can clearly classify RBC morphology whether normal or abnormal. Some data are still overlapped but those data are not applicable to use mean and SD to differentiate. Though, there are some parameters such as WBC, Hb, Hct, RBC, MCV, MCH, MCHC, and PLT that are significantly different. According to what we found, Hb, Hct, RBC, MCV, MCH, and MCHC are all indication of overall red blood cell, so the variations in those parameters are related to the variation of changes in red blood cell morphology. MCV, MCH, Hct, and RBC are related to changes found in RBC morphology on blood smear more than those found in RBC and MCHC. Hence, when analyzed with J48 algorithm, we found these parameters on decision tree arranged in order of degree of changes in RBC morphology on blood smear (MCV, MCH, Hct, and RBC, resp.). Whichever a parameter that has minimal or no effect is not specific to RBC morphology as seen in MCHC. Though MCHC is the parameter that reflects mean corpuscular hemoglobin concentration of individual red blood cell, report from previous studies has shown that MCHC showed the least change but the use of MCHC is limited to only in quality control purpose more than in diagnostic purpose [6]. WBC and PLT are differently found in normal and abnormal RBC morphology which is expected to see in other parameters. This occurrence is unsupported by any previous studies, so it is only an occasional agreement.

This study has shown the benefit of applying data mining technique by creating a practical guideline in blood smear examination from decision tree (as seen in Figure 1). An advantage of a decision tree is to assist in decision making. That is the number appears in decision tree is an exactly value which is easier to make a decision than using a normal range in laboratory, thus it will reduce uncertainty in decision making. Moreover, it can be used as a practical guideline for RBC morphology from hematological parameter. However, what we have shown is only a practical guideline, however scanning a peripheral blood smear is encouraged. In addition, due to the limitation of samples, we have only normal and microcytic red blood cell in thalassemia and hemoglobinopathies which a common anemia in Thailand [7]. The study did not cover macrocytic anemia, because of it low percentage and only found in older Thai. In the future, data collection should include macrocytic anemia in order to make the complete practical guideline for clinical laboratory improvement.

## 5. Conclusion

Data mining by using J48 algorithm shows a distinctive point of data mining in analyzing a relationship of a complex data that exceeds capability of a common statistics. The J48 is a mathematic calculation cooperates with simulation model and a simple decision tree in order to create a practical guideline in predicting RBC morphology from hematological parameters of 4 data sets (MCV, MCH, Hct, and RBC) from 13 datasets which can be used for RBC morphology from hematological parameters such as teenager for baseline data.

## Figures and Tables

**Table 1:** The data set in this study.

Number	Code	Description	Domain
1	Sex	Sex	Male, female
2	WBC	White blood cell count (cell/uL)	Integer
3	RBC	Red blood cell count	Integer
4	Hb	Hemoglobin (g/dL)	Integer
5	Hct	Hematocrit (%)	Integer
6	MCV	Mean corpuscular volume (fL)	Integer
7	MCH	Mean corpuscular hemoglobin (pg)	Integer
8	MCHC	Mean corpuscular hemoglobin concentration (g/dL)	Integer
9	PLT	Platelet count (cell/uL)	Integer
10	NEU	Neutrophil count (%)	Integer
11	LYMP	Lymphocyte count (%)	Integer
12	MONO	Monocyte count (%)	Integer
13	EO	Eosinophil count (%)	Integer
14	BASO	Basophil count (%)	Integer
15	RBC morphology	Red blood cell morphology	Normal, abnormal

**Table 2:** The hematological parameters which were categorized by RBC morphology and sex.

Hematological parameters	Female			Male		
	Abnormal blood smear (mean ± SD)	Normal blood smear (mean ± SD)	Significant (*P* < 0.05)	Abnormal blood smear (mean ± SD)	Normal blood smear (mean ± SD)	Significant (*P* < 0.05)
WBC (×10^3^cell/µL)	7.87 ± 1.85	7.62 ± 1734.89	S	8.27 ± 2.07	7.31 ± 1.66	S**
RBC (cell/µL)	5.28 ± 0.56	4.73 ± 0.36	S	6.41 ± 0.58	5.45 ± 0.38	S
Hb (mg/dL)	11.64 ± 1.22	13.02 ± 0.83	S	13.86 ± 1.11	15.34 ± 0.86	S
Hct (%)	36.18 ± 3.33	40.03 ± 2.41	S	42.66 ± 2.89	46.17 ± 2.35	S
MCV (fL)	69.09 ± 7.45	84.9 ± 4.93	S	66.98 ± 6.55	84.88 ± 4.53	S
MCH (pg)	22.22 ± 2.56	27.62 ± 1.78	S	21.76 ± 2.41	28.21 ± 1.64	S
MCHC (g/dL)	32.16 ± 1.36	32.53 ± 0.76	S	32.48 ± 1.32	33.23 ± 0.72	S
PLT (×10^3^ cell/µL)	313.85 ± 69.64	273.12 ± 54.92	S	282.08 ± 61.59	253.15 ± 47.35	S
NEU (%)	57.09 ± 8.23	57.22 ± 8.06	NS	59.67 ± 9.16	57.54 ± 7.85	NS*
LYMP (%)	37.57 ± 7.58	37.45 ± 7.41	NS	34.89 ± 8.26	36.83 ± 6.98	NS
MONO (%)	2.96 ± 1.03	2.99 ± 1.05	NS	2.67 ± 0.93	2.88 ± 1.06	NS
EO (%)	2.18 ± 1.95	2.12 ± 1.93	NS	2.51 ± 1.9	2.49 ± 2.45	NS
BASO (%)	0.2 ± 0.4	0.22 ± 0.41	NS	0.27 ± 0.45	0.26 ± 0.44	NS

Total	318	784		36	224	

*NS: no statistically significant; **S: statistically significant.

**Table 3:** The confusion matrix of predicted RBC morphology by J48 algorithm.

Actual class from manual RBC morphology report	Predicted class from J48 model	
	Abnormal	Normal
Abnormal	TP (338)	FN (16)
Normal	FP (66)	TN (942)

**Table 4:** The performance evaluation of predicted normal and abnormal RBC morphology.

Class	Abnormal	Normal	Average
TP	0.955	0.935	0.940
FP	0.065	0.045	0.050
Precision	0.837	0.983	0.945
Recall	0.955	0.935	0.940
*F*-Measure	0.892	0.958	0.941
Accuracy	0.943	0.943	0.943
